# Enzymatic upcycling of wild-simulated ginseng leaves for enhancing biological activities and compound K

**DOI:** 10.1007/s00253-024-13028-2

**Published:** 2024-02-14

**Authors:** Juho Lim, Hayeong Kim, Gha-hyun J. Kim, Taeyoon Kim, Choon Gil Kang, Seung Wook Kim, Doman Kim

**Affiliations:** 1 Graduate School of International Agricultural Technology, Seoul National University, Pyeongchang-gun, Gangwon-do 25354 Republic of Korea; 2 Institute of Food Industrialization, Institutes of Green Bioscience & Technology, Center for Food and Bioconvergece, Seoul National University, Pyeongchang-gun, Gangwon-do 25354 Republic of Korea; 3 Department of Bioengineering and Therapeutic Sciences and Programs in Biological Sciences and Human Genetics, University of California, San Francisco, CA 94158 USA; 4 Ottogi Corporation, Anyang-si, Gyeonggi-do 14060 Republic of Korea; 5 Fervere Campus Corporation, Pyeongchang-gun, Gangwon-do 25354 Republic of Korea

**Keywords:** Wild-simulated ginseng leaves, Minor ginsenosides, Enzymatic biotransformation, Compound K, Anti-senescence, Anti-inflammation

## Abstract

Compound K (CK), a ginsenoside with high bioavailability, is present at low levels in wild-simulated ginseng leaves (WSGL). WSGL contains the CK precursors, Rd and F2, in amounts up to 26.4 ± 0.4 and 24.1 ± 1.9 mg/g extract, respectively. In this study, CK production in WGSL reached 25.9 ± 1.0 mg/g extract following treatment with Viscozyme, Celluclast 1.5 L, Pectinex Ultra SP-L, and their combination. The antioxidant activities indicated by oxygen radical absorbance capacity, ferric reducing antioxidant power, and ABTS- and DPPH radical scavenging activity of enzyme-treated WSGL were enhanced 1.69-, 2.51-, 2.88-, and 1.80-fold, respectively, compared to non-treated WSGL. Furthermore, the CK-enriched WSGL demonstrated a 1.94-fold decrease in SA-β-galactosidase expression in human dermal fibroblasts and a 3.8-fold enhancement of inhibition of nitric oxide release in lipopolysaccharide-induced RAW 264.7 cells relative to non-treated WSGL. Consequently, WSGL subjected to enzymatic upcycling has potential as a functional material in the food and pharmaceutical industries.

**Supplementary Information:**

The online version contains supplementary material available at 10.1007/s00253-024-13028-2.

## Key points

• *Food-grade commercial enzymes were used to produce compound K in wild-simulated ginseng leaves*.

• *Compound K of WSGL was produced up to 25.9 mg/g extract by enzyme treatment*.

• *CK-enriched WSGL inhibited SA-β-galactosidase expression and nitric oxide release in cells*.

**Supplementary Information:**

The online version contains supplementary material available at 10.1007/s00253-024-13028-2.

## Introduction

Korean ginseng, *Panax ginseng* C. A. Mey, is used in herbal medicine and dietary supplements because of its medicinal properties (Chang et al. 2003; Lim et al. 2023). Ginseng cultivated in the mountains without artificial intervention for over 5 years is termed wild-simulated ginseng (WSG). According to the Korean Ministry of Food and Drug Safety (2022), the leaves, fruits, and roots of WSG can be used as food materials. WSG leaves (WSGL) have a ginsenoside composition similar to *P. ginseng* roots, such as Rb1, Rc, Re, and Rd. However, the total ginsenoside content in WSGL is 1.92-fold higher than that in field-cultivated ginseng. WSG is over 28 times more expensive than field-cultivated ginseng due to its lower survival rate and smaller size. This high cost hinders its use as an ingredient in the nutraceutical and pharmaceutical industries (Sheban et al. 2022). Unlike the roots, WSGL can be harvested annually, making it a potential source of materials for industrial applications.

Compound K (CK; 20-O-β-D-glucopyranosyl-20(S)-protopanaxadiol) is a minor tetracyclic triterpenoid with neuroprotective, anti-aging, antidiabetic, anticancer, anti-inflammatory, anticarcinogenic, and antiallergic activities (Cho et al. 2009; Han et al. 2007; Jeong et al. 2010; Kim et al. 2004; Park et al. 2005; Shin et al. 2005). However, CK is present in trace amounts or is non-detectable in WSG and red ginseng (Lee et al. 2020; Lim et al. 2023; Park et al. 2021) and is typically produced from major ginsenosides by chemical treatment, microbial fermentation, or enzymatic biotransformation (Han et al. 1982; Kim et al. 2018; Lee et al. 2020). Enzymatic biotransformation has high specificity, purity, and simplicity (Kim et al. 2021; Upadhyaya et al. 2016). The biotransformation pathway for CK from major ginsenosides is theorized to be as follows: Rb1, Rc, Rb2 → Rd → Rg3, F2 → CK (Sharma and Lee 2020). However, the Rd and F2 contents of WSG root were 1.28 and 0.11 mg/g dry weight (DW) and 5.16 and 2.45 mg/g extract in red ginseng, compared to 5.45 and 10.85 mg/g DW in WSGL (Lee et al. 2020; Lim et al. 2023; Park et al. 2021), making WSGL a more suitable material for producing CK by enzymatic transformation. Food-grade commercial enzymes, such as cellulase, hemicellulase, pectinase, amylase, β-glucosidase, polygalacturonase, and glucanase, which are used to break glycosidic linkages, have been employed to produce minor ginsenosides, including CK (Song et al. 2017; Choi et al. 2014). Mok et al. (2023) showed that CK was produced at 0.03 mg/g from WSG using Pectinex Ultra SP-L (PecS) under 100 MPa pressure (Mok et al. 2023). Park et al. (2021) reported that red ginseng had a CK content of 2.47 mg/g extract after treatment with Sumizyme AC for 48 h, which increased to 14.32 mg/g after treatment with a combination of Ultimase MFC, Pyr-flo, and Rapidase for the same duration (Park et al. 2021). Moreover, cultivated ginseng leaves have been treated with several commercial enzymes, including Celluclast 1.5 L (Cel), Cytolase PCL5, Econase CE, and Optidex L-400. Of these, Cytolase PCL5 yielded CK at 8.73 mg/g (Lee et al. 2012). However, comprehensive studies on the effects of commercial enzymes and their combinations on CK production from WSGL and their biochemical properties are limited. Herein, CK was produced in WSGL, and the F1 and F2 contents in WSGL were enhanced using Vis, Cel, PecS, and their combination. The antioxidant activity of WSGL-enriched CK was analyzed by performing oxygen radical antioxidant capacity (ORAC), ferric-reducing antioxidant power (FRAP), and ABTS- and DPPH radical scavenging activity assays. Furthermore, the effects of enzyme-treated WSGL-enriched CK on SA-β-galactosidase in human dermal fibroblasts (HDFs) and nitric oxide (NO) release by lipopolysaccharide (LPS)-stimulated RAW264.7 macrophages were analyzed. This work provides a basic method for producing CK and other minor ginsenosides, such as Rh1, F1, and F2, using commercial enzymes in combination.

## Materials and methods

### Materials

WSGL were obtained from a farm in Pyeongchang (Gangwon-do, South Korea). Ginsenoside standards (Rb1, Rc, Rd, Re, Rg1, Rg2, Rg3, Rh1, F1, F2, and CK) were purchased from ChemFaces (Wuhan, China). Dimethyl sulfoxide (DMSO), 2,2-diphenyl-1-picrylhydrazyl (DPPH), folin–ciocaulteu reagent, 2,2′-azobis(2-methylpropionmidine) dihydrochloride (AAPH), 6-hydroxy-2,5,7,8-tetramethylchromane-2-carboxylic acid (Trolox), 2,2’-azinobis-(3- ethylbenzothiazoline-6-sulfonic acid) (ABTS), aluminum chloride, and gallic acid (GA) were from Sigma-Aldrich (St. Louis, MO, USA). Potassium acetate was purchased from Duksan Chemicals (Seoul, Korea). Fluorescein was sourced from Alfa Aesar (Haverhill, MA). RAW264.7 murine macrophages and HDFs were purchased from the Korean Cell Line Bank (KCLB, Seoul, Korea) and American Type Culture Collection (ATCC, Manassas, VA, USA), respectively. Dulbecco’s modified Eagle’s medium (DMEM), and fetal bovine serum (FBS) were obtained from Gene Depot (Barker, TX, USA), and penicillin and streptomycin (PS) were purchased from Invitrogen (Carlsbad, CA, USA). Ez-CyTox solution for cell viability assay was supplied by Daeil Lab Service (Seoul, Korea). *Saccharomyces cerevisiae* was obtained from Jenico Inc. (Seoul, Korea). Viscozyme (Vis: 100 β-glucanase U/mL), Celluclast 1.5L (Cel: 700 endo glucanase U/mL), and Pectinex ultra SP-L (PecS: 3,800 polygalactorunase U/mL) were purchased from Novozymes (Bagsværd, Denmark).

### Extraction of WSGL

WSGL (20 g) was added to 1 L of 70% (v/v) ethanol, sonicated using a KUS-1200 ultrasonic homogenizer (Korea Bio Tech, Seong-Nam, Korea), and placed in a 60 °C water bath for 60 min. Subsequently, the supernatant was centrifuged at 9,600 × g for 20 min at 4 °C and filtered using Whatman No. 1 filter paper (Piscataway, NJ, USA). The pellets were re-extracted three times as described above. Ethanol was eliminated by evaporation (Heidolph Instruments, Schwabach, Germany) at 55 °C. The monosaccharides and disaccharides in the extracted WSGL solution were removed by adding sodium alginate yeast beads to the supernatants (Duarte et al. 2013). The sample was incubated at 37 °C and 150 rpm for 12 h. The yeast beads were removed, and the supernatant was lyophilized at 0 °C and 10 Pa (Eyela FD-550; Rikakikai Co., Tokyo, Japan) for analysis of ginsenoside composition as described previously (Lim et al. 2023). After lyophilization, a 20 mg sample was dissolved in DMSO as a stock solution. Samples were diluted with methanol and filtered through a 0.2-µm membrane syringe filter (Hyundai Micro, Seoul, Korea). Next, 20 µL of diluted sample was injected into a BEH C_18_ column (2.1 × 150 mm, 1.7 μm; Waters, Milford, MA, USA) connected to an ultra-performance liquid chromatography-mass spectrometry (UPLC-MS, Acquity H-Class, Waters) system with a quadrupole Dalton (QDa) detector at a flow rate of 0.3 mL/min. Acetonitrile containing 0.1% formic acid (solvent A) and water containing 0.1% formic acid (solvent B) were used as the mobile phases; the elution gradient was 0–2.0 min 33% A, 2.0–9.0 min 38% A, 9.0–13.0 min 58% A, 13.0–15.0 min 77% A, 15.0–18.0 min 100% A, 18.0–20.0 min 100% A, and 20.0–25.0 min 5% A. Ginsenosides at concentrations of 0.1–20.0 µg/mL were used as standards (Table s1).

### Production of minor ginsenosides using commercial enzyme

For single commercial enzyme treatments, WSGL was treated with Vis, Cel, or PecS. This involved adding 5% (v/v) of the enzyme to a reaction mixture that contained 50 mg WSGL/mL in a 20 mM sodium acetate buffer (Na-Ac) (pH 5.2). The mixture was then incubated at 50 °C for either 24 or 48 h. For combined enzyme treatments (Vis + Cel, Vis + PecS, or Cel + PecS), WSGL was similarly treated. Here, 5% (v/v) of Vis, PecS, or Cel was added to the reaction mixture containing 50 mg WSGL/mL and an additional 5% (v/v) of Cel, Vis, or PecS in 20 mM Na-Ac (pH 5.2). This mixture was also reacted at 50 °C for 24 h or 48 h. The reaction was halted by boiling the mixture at 100 °C for 5 min. The sample was then lyophilized at 0 °C and 10 Pa. Ginsenoside composition was analyzed as previously described. The WSGL treated for 48 h was selected for further study.

### Determination of total saponin, phenolic, and flavonoid content of enzyme-treated WSGL

#### Total saponin content (TSC)

The total saponin content in WSGL and enzyme-treated WSGL was analyzed using the vanillin-sulfuric acid method, with ginsenoside Re as the standard. In brief, the sample was placed into a 96-well plate along with 8% (w/v) vanillin and 72% (v/v) sulfuric acid, mixed at a ratio of 1:1:10 (v/v/v). This mixture was then incubated at 60 °C for 10 min, followed by cooling on ice for 5 min. Absorbance was measured at 544 nm using a SpectraMax M3 microplate reader (Molecular Devices, Sunnyvale, CA). The total saponin content (TSC) was expressed as mg of Re per g of extract (mg Re/g extract).

#### Total phenolic content (TPC)

The total phenolic content of WSGL and enzyme-treated WSGL was determined using the folin-ciocalteu method with GA as the standard. Briefly, 150 µL of the sample or the standard (0 − 100 µM) was added to a 96-well plate containing 15 µL of folin-ciocalteu reagent and vortexed for 3 min in a dark condition. Next, 15 µL of 10% (w/v) Na_2_CO_3_ was added, and the mixture was reacted at 22 °C in the dark condition. After 30 min, the plate was measured at 760 nm using a SpectraMax M3 microplate reader. TPC was expressed as mM gallic acid equivalent (GAE)/g extract (mM GAE/g extract).

#### Total flavonoid content (TFC)

The total flavonoid content of WSGL and enzyme-treated WSG was analyzed using the aluminum chloride method with quercetin as the standard. Briefly, 180 µL of the sample or the standard (0‒1,000 µM) was added to a 96-well plate containing 10 µL of 10% (w/v) aluminum chloride and 1 M potassium acetate. The mixture was incubated at 28 °C for 30 min and followed by reading at 415 nm using a SpectraMax M3 microplate reader. TFC was expressed as mM quercetin equivalent (QE)/g extract (mg QE/g extract).

### Analysis of the antioxidant properties of enzyme-treated WSGL

#### Oxygen radical absorbance capacity (ORAC)

ORAC assay of non-treated and enzyme-treated WSGL was carried out as described previously (Lim et al. 2023). Fluorescein was used as the probe and AAPH was used to induce radical formation. The fluorescence of the reaction was measured every 3 min (λ_excitation_ = 485 nm, λ_emission_ = 538 nm) for 2 h at 37 °C using the SpectraMax M3 microplate reader. ORAC values are expressed as mM Trolox equivalent (TE)/g extract (mM TE/g extract).

#### Ferric reducing antioxidant power (FRAP)

FRAP solution was prepared by mixing 0.3 M Na-Ac (pH 3.6), 10 mM 2,4,6-tripyridyl-s-triazine, and 20 mM ferric chloride hexahydrate solution at a ratio of 1:10:1 (v/v/v). The FRAP assay was started by adding 20 µL of the sample or ferrous sulfate heptahydrate (1–2,500 µM) with 180 µL of FRAP solution in a 96-well plate followed by incubation for 30 min. The plate was read at 593 nm using the SpectraMax M3 microplate reader. FRAP values are expressed as mM Fe^2+^/g extract.

#### DPPH radical scavenging activity

The DPPH radical scavenging activities of non-treated and enzyme-treated WSGL were determined by mixing 0.1 mM DPPH with samples or Trolox standard (0–250 µM) in a 96-well plate. The reaction mixture was incubated at 28 °C for 30 min. The plate was read at 517 nm by the SpectraMax M3 microplate reader. DPPH radical scavenging activity is expressed as mM TE/g extract.

#### ABTS radical scavenging activity

ABTS solution was prepared by mixing 7 mM ABTS and 2.45 mM potassium persulfate in 75 mM sodium phosphate buffer (pH 7.4) and keeping it at 22 °C for 12 h. Then, the solution was filtered using a 0.45 μm syringe filter (Satoris, Goettingen, Germany) and diluted with buffer until the absorbance was 0.70 ± 0.02 at 734 nm. For the ABTS radical scavenging activity assay, 180 µL of ABTS solution was mixed with 20 µL of the sample or Trolox standard (0–400 µM) in a 96-well plate. The plate was incubated at 28 °C. After 10 min, the plate was read at 734 nm by the SpectraMax M3 microplate reader. Reaction with sodium phosphate buffer and without sample was used as control. ABTS radical scavenging activity is expressed as mM TE/g extract.

### Cell viability assay

HDFs and RAW264.7 murine macrophages were cultured in DMEM containing 10% (v/v) FBS, 100 U/mL penicillin, and 100 µg/mL streptomycin at 37 °C in 5% CO_2_ until 70–80% confluence. Cells were dispensed at 6 × 10^4^/mL in a 96-well plate and incubated at 37 °C under 5% CO_2_ for 24 h, followed by adding non-treated and enzyme-treated WSGL (50–400 µg/mL). After 24 h, 100 µL of solution composed of 90 µL of culture medium and 10 µL of Ez-CyTox solution was added to each well, followed by incubation for 1 h at 37 °C. The plate was measured at 450 nm using a SpectraMax M3 microplate reader. Cell viability is expressed as a percentage of the control.

### Anti-senescence effect of enzyme-treated WSGL

HDFs were seeded at 5 × 10^4^/mL in a six-well plate and incubated at 37 °C in 5% CO_2_ for 24 h. The cells were added to 50 µg/mL non-treated and enzyme-treated WSGL for 24 h, followed by treatment with 0.1 µM doxorubicin for 48 h. The medium was exchanged for DMEM containing 10% (v/v) FBS, 100 U/mL penicillin, and 100 µg/mL streptomycin followed by incubation for 72 h. The medium was removed, and cells were washed with phosphate-buffered saline, fixed with 3.7% (v/v) formaldehyde for 15 min, and stained with X-gal staining solution (5 mM potassium ferricyanide, 5 mM ferrocyanide, 150 mM sodium chloride, 2 mM magnesium chloride, and 1 mg/mL X-gal) for 24 h. SA-β-gal activity was visualized using an Observer Z1 microscope (Carl Zeiss, Jena, Germany) and photographed using an AxioCam HRc camera. The level of SA-β-gal activity was determined by randomly selecting fields and analyzing the signal intensity using ImageJ software (National Institutes of Health, Bethesda, MD, USA). The level of SA-β-gal activity is described as a percentage of the positive control.

### Inhibition of NO production

RAW264.7 murine macrophages were seeded in a 96-well plate at 2 × 10^4^/well and incubated at 37 °C under 5% CO_2_. After 24 h, the cells were treated with 100 µg/mL non-treated and enzyme-treated WSGL and 1 µg/mL LPS for 24 h. Cells treated with 1 µg/mL LPS or 1 µg/mL LPS and 100 µM indomethacin were used as the negative and positive controls, respectively. NO release was determined by mixing 80 µL of Griess reagent and 80 µL of culture supernatant in a 96-well plate at room temperature for 20 min and reading at 540 nm using the SpectraMax M3 microplate reader.

### Statistical analysis

Results are means ± standard deviation (*n* = 3). Differences between groups were subjected to *t*-test and one-way analysis of variance (ANOVA) followed by Duncan’s multiple range test using SPSS software (v. 26.0; IBM Corp., Armonk, NY, USA). A value of *p* < 0.05 was considered indicative of statistical significance. Prism software (v. 8.0; GraphPad Software Inc., San Diego, CA, USA) was used to generate plots and ChemDraw software (v. 22.2.0; PerkinElmer Inc., Waltham, MA, USA) was used to draw ginsenoside structures.

## Results

### Ginsenoside composition in extracted WSGL

**Fig. 1:**
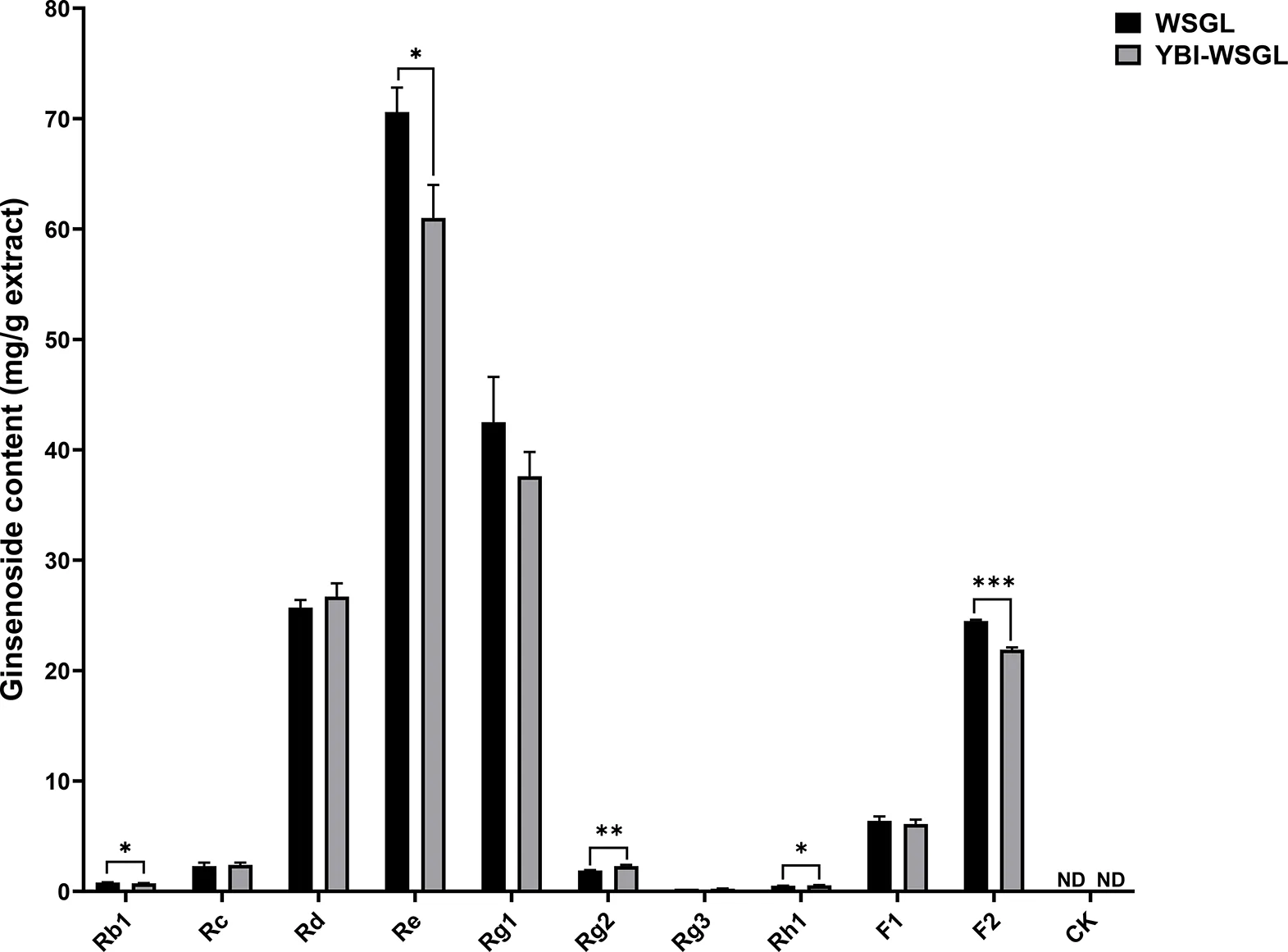
Ginsenoside composition changes by yeast bead immobilization. WSGL: wild-simulated ginseng leaves; YBI-WSGL: wild-simulated ginseng leaves after yeast bead immobilization. Values were expressed as means ± standard deviation (SD) of three independent experiments. Asterisks (*, ** and ***) indicate a significant difference (*: *p* < 0.05; **: *p* < 0.01; ***: *p* < 0.001)

The extraction yield of WSGL was 41.5 ± 1.4%. The ginsenoside contents of WSGL extracted using UPLC-MS are listed in Fig. 1. Of the 11 ginsenoside standards, 10 were detected in extracted WSGL by UPLC-MS, the exception being CK. Five major ginsenosides (Rb1, Rc, Rd, Re, and Rg1) in extracted WSGL constituted 80.9% of the total extracted ginsenosides. Rb1 and Rc were present at low concentrations, comprising < 1.5% of the total extracted ginsenosides. The ginsenoside with the highest content in WSGL was Re, followed by Rg1 and Rd (Fig. 1). In this study, five minor ginsenosides (Rg2, Rg3, Rh1, F1, and F2) were detected in extracted WSGL, constituting 19.1% of the total ginsenosides.

### Effect of yeast bead immobilization on ginsenoside contents

The contents of fructose, glucose, and sucrose in extracted WSGL were 40.1 ± 1.1, 77.9 ± 0.4, and 14.0 ± 1.3 mg/g extract, respectively. After yeast bead immobilization, glucose, fructose, and sucrose were not detected in the extracted WSGL. The effect of yeast bead immobilization on ginsenoside composition is shown in Fig. 1. The total ginsenoside content after yeast bead immobilization represented 90.9 ± 3.3% of the total ginsenosides in extracted WSGL. After yeast bead immobilization, Rb1, Re, Rg2, Rh1, and F2 were reduced (Fig. 1), while Rc, Rd, Rg1, Rg3, and F1 were non-significant differences between before and after yeast bead immobilization.

### Effect of single enzyme treatment on the production of minor ginsenosides in WSGL

**Fig. 2:**
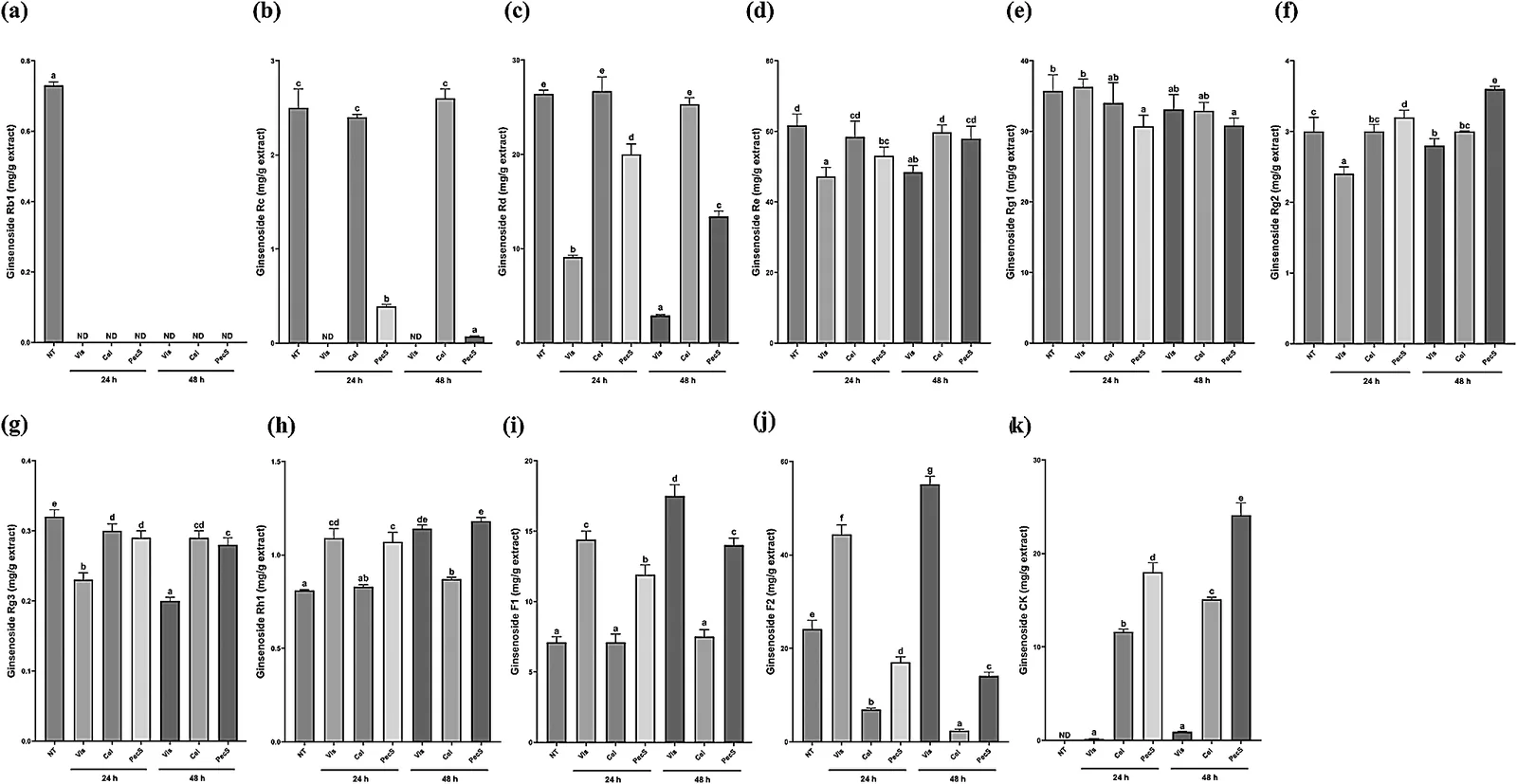
The contents of ginsenoside Rb1 (**a**), Rc (**b**), Rd (**c**), Re (**d**), Rg1 (**e**), Rg2 (**f**), Rg3 (**g**), Rh1 (**h**), F1 (**i**), F2 (**j**), compound K (**k**) of WSGL treated by Vis, Cel, and PecS. NT: non-treated; Vis, Cel, PecS: single enzyme-treated WSGL; Vis + Cel, Vis + PecS, Cel + PecS: combination of enzyme-treated WSGL. Values were expressed as means ± standard deviation (SD) of three independent experiments. Different letters indicate statistical differences by Duncan’s test

In this study, three commercial enzymes, including Vis and PecS from *Aspergillus aculeatus* and Cell from *Trichoderma reesei*, were used for the treatment of WSGL (Table s2). These enzymes were worked in a pH range of 3.5 − 6.0 and temperature ranges of 25 − 65 °C. The ginsenoside composition in enzyme-treated WSGL is presented in Fig. 2. Vis-treated WSGL showed complete hydrolysis of Rb1 and Rc, and its Rd, Re, Rg2, and Rg3 contents were decreased by 2.9-, 1.31-, 1.25-, and 1.3-fold after 24 h and by 9.1-, 1.27-, 1.14-, and 1.6-fold after 48 h, respectively, compared to non-treated WSGL (Fig. 2a–g). By contrast, the Rh1, F1, and F2 contents were increased 1.35-, 2.03-, and 2.47-fold after treatment for 24 h and by 1.41-, 1.84-, and 2.29-fold after 48 h compared to non-treated WSGL (Fig. 2h–j). Cel-treated WSGL showed no significant differences in the contents of Rc, Rd, Re, Rg1, Rg2, Rg3, Rh1, or F1, but Rb1 was completely hydrolyzed (Fig. 2a–i). Moreover, the F2 content in Cel-treated WSGL decreased 3.49-fold after treatment for 24 h and 10.04-fold after 48 h compared to non-treated WSGL (Fig. 2j). PecS-treated WSGL exhibited complete hydrolysis of Rb1 (Fig. 2a). The Rc, Rd, Rg1, and F2 contents decreased 6.41-, 1.32-, 1.16-, and 1.42-fold after treatment for 24 h, and 36.43-, 1.97-, 1.16-, 1.70-fold after 48 h compared to non-treated WSGL (Fig. 2b, c, e, j). Additionally, the Rh1 and F1 contents increased 1.32- and 1.68-fold after treatment for 24 h, and 1.46- and 1.97-fold after 48 h, respectively, compared to non-treated WSGL (Fig. 2h, i). The CK was produced in enzyme-treated WSGL at 0.17–18.0 mg/g extract after treatment for 24 h and 0.91–24.1 mg/g extract after treatment for 48 h (Fig. 2k). CK of WSGL was produced at 0.17 ± 0.03 mg/g extract by Vis treatment, and increased to 11.6 ± 0.3 and 15.1 ± 0.2 mg/g extract by Cel and PecS treatment, respectively (Fig. 2k). The highest CK content was in PecS-treated WSGL.

### Effects of enzyme combinations on the contents of minor ginsenosides in WSGL

**Fig. 3:**
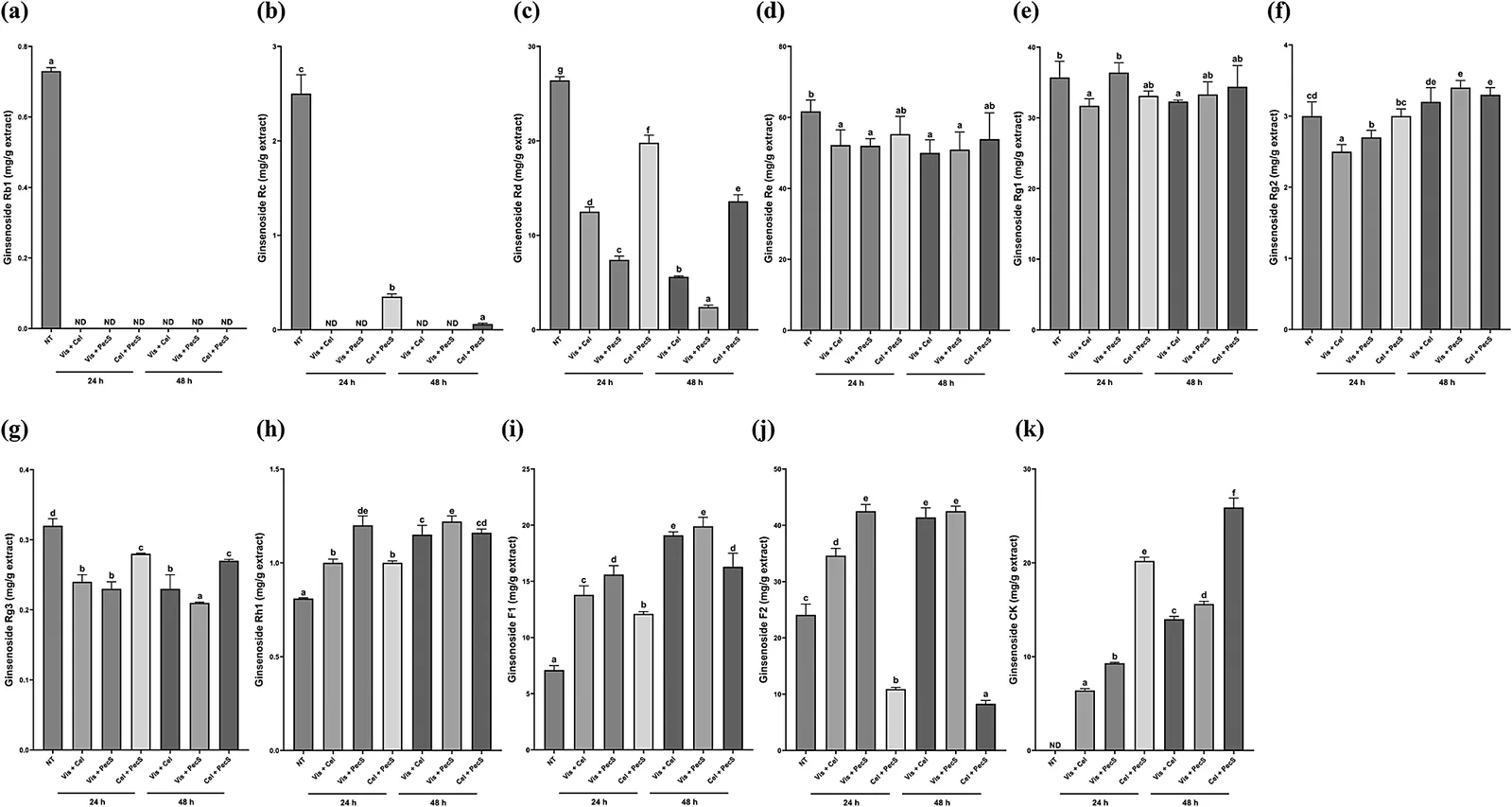
The contents of ginsenoside Rb1 (**a**), Rc (**b**), Rd (**c**), Re (**d**), Rg1 (**e**), Rg2 (**f**), Rg3 (**g**), Rh1 (**h**), F1 (**i**), F2 (**j**), compound K (**k**) of WSGL treated by the combination of Vis + Cel, Vis + PecS, and Cel + Pecs. NT: non-treated; Vis, Cel, PecS: single enzyme-treated WSGL; Vis + Cel, Vis + PecS, Cel + PecS: combination of enzyme-treated WSGL. Values were expressed as means ± standard deviation (SD) of three independent experiments. Different letters indicate statistical differences by Duncan’s test

The effects of Vis + Cel, Vis + PecS, and Cel + PecS combinations on the production of minor ginsenosides are shown in Fig. 3. Vis + Cel completely hydrolyzed Rb1 and Rc in WSGL extract, and decreased the Rd, Re, Rg1, and Rg3 contents in WSGL extract by 2.11-, 1.18-, 1.2-, and 1.33-fold after 24 h and 4.71-, 1.22-, 1.11-, and 1.39-fold after 48 h, respectively, compared to non-treated WSGL (Fig. 3a-g). By contrast, the F1 contents of Vis + Cel-treated WSGL increased from 7.1 ± 0.04 to 19.1 ± 0.03 mg/g extract, and that of F2 increased from 24.1 ± 1.90 to 41.4 ± 0.03 mg/g extract (Fig. 3i, j). The CK content in Vis + Cel-treated WSGL increased to 6.4 ± 0.2 mg/g extract after 24 h and 14.0 ± 0.3 mg/g extract after 48 h (Fig. 3k). There was no significant difference in F1 content between Vis- and Vis-Cel treatment for 24 h. The F1 content in Vis + Cel-treated WSGL after 48 h was 1.38-fold higher than at 24 h. The F2 content in WSGL was not significantly different between Vis treatment after 24 h and Vis + Cel treatment after 48 h, and the F2 content in Vis + Cel-treated WSGL after 24 h was decreased 1.28- and 1.59-fold compared to Vis-treated WSGL after 24 and 48 h, respectively. The F2 content in Vis + Cel-treated WSGL after 48 h was increased 1.20-fold compared to 24 h but decreased 1.33-fold compared to Vis-treated WSGL after 48 h. The Rh1 content did not differ significantly between Vis- and Vis + Cel-treated WSGL. However, the CK content in Vis + Cel-treated WSGL was increased from 0.17 ± 0.03 to 6.4 ± 0.2 mg/g extract after 24 h and from 0.17 ± 0.03 tract to 14.0 ± 0.3 mg/g extract after 48 h (Fig. 3k). Therefore, Cel in combination with Vis contributed to the conversion of F2 to CK in WSGL.

After Vis + PecS treatment, Rb1 and Rc in extracted WSGL were completely hydrolyzed, whereas the Rd, Re, and Rg3 contents in Vis + PecS-treated WSGL were decreased by 3.57-, 1.19-, and 1.39-fold after 24 h and 11.0-, 1.21-, and 1.52-fold after 48 h (Fig. 3a-d, g). By contrast, the F1 content of Vis + PecS-treated WSGL increased from 7.1 ± 0.04 to 19.9 ± 0.80 mg/g extract, and that of F2 increased from 24.1 ± 1.90 to 42.5 ± 0.90 mg/g extract. The F2 content in Vis + PecS-treated WSGL differed depending on whether the treatment was carried out for 24 or 48 h (Fig. 3j). The Rh1 content in Vis + PecS-treated WSGL was increased 1.48- and 1.51-fold after 24 and 48 h, respectively (Fig. 3h). Although the CK content in Vis + PecS-treated WSGL increased to 15.6 ± 0.30 mg/g extract after 48 h, it was 1.94- and 1.54-fold lower than that of PecS-treated WSGL after 24 and 48 h, respectively (Figs. 2k and 3k). However, the Vis + PecS combination increased the CK content 1.45- and 1.11-fold after 24 and 48 h, respectively, compared to Vis + Cel-treated WSGL (Fig. 3k). The F1 and F2 contents in Vis + PecS-treated WSGL were 1.31–1.42- and 2.50‒3.01-fold higher than those in PecS-treated WSGL (Figs. 2i–j and 3i–j). Therefore, the treatment of WSGL with the combination of Vis and PecS resulted in increased contents of CK, F1, and F2.

The treatment of WSGL with the combination of Cel + PecS results in hydrolysis of Rb1, similar to Vis-, Cel-, PecS-, Vis + Cel-, and Vis + PecS-treated WSGL. The Rc, Rd, Rg3, and F2 contents in Cel + PecS-treated WSGL were decreased by 7.14-, 1.33-, 1.14-, and 2.21-fold after 24 h and 41.67-, 1.94-, 1.19-, and 2.90-fold after 48 h, respectively (Fig. 3a–c, g, j). The Rh1 content in Cel + PecS-treated WSGL increased 1.25- and 1.43-fold after 24 and 48 h (Fig. 3h). By contrast, the F1 content in Cel + PecS-treated WSGL increased from 7.1 ± 0.04 to 16.30 ± 1.20 mg/g extract (Fig. 3i). Although the F1 content did not differ significantly between PecS- and Cel + PecS-treated WSGL after 24 h, it was 1.70-fold higher in Cel + PecS-treated compared to Cel-treated WSGL. Moreover, the CK content in Cel + PecS-treated WSGL increased to 20.2 ± 0.40 mg/g extract after 24 h and 25.9 ± 1.0 mg/g extract after 48 h (Fig. 3k). These results revealed that the minor ginsenoside contents of enzyme-treated WSGL after 48 h was higher than that of enzyme-treated WSGL after 24 h. Therefore, enzyme-treated WSGL after 48 h was selected for further study.

### Determination of total saponin, phenolic, and flavonoid content of enzyme-treated WSGL

**Fig. 4:**
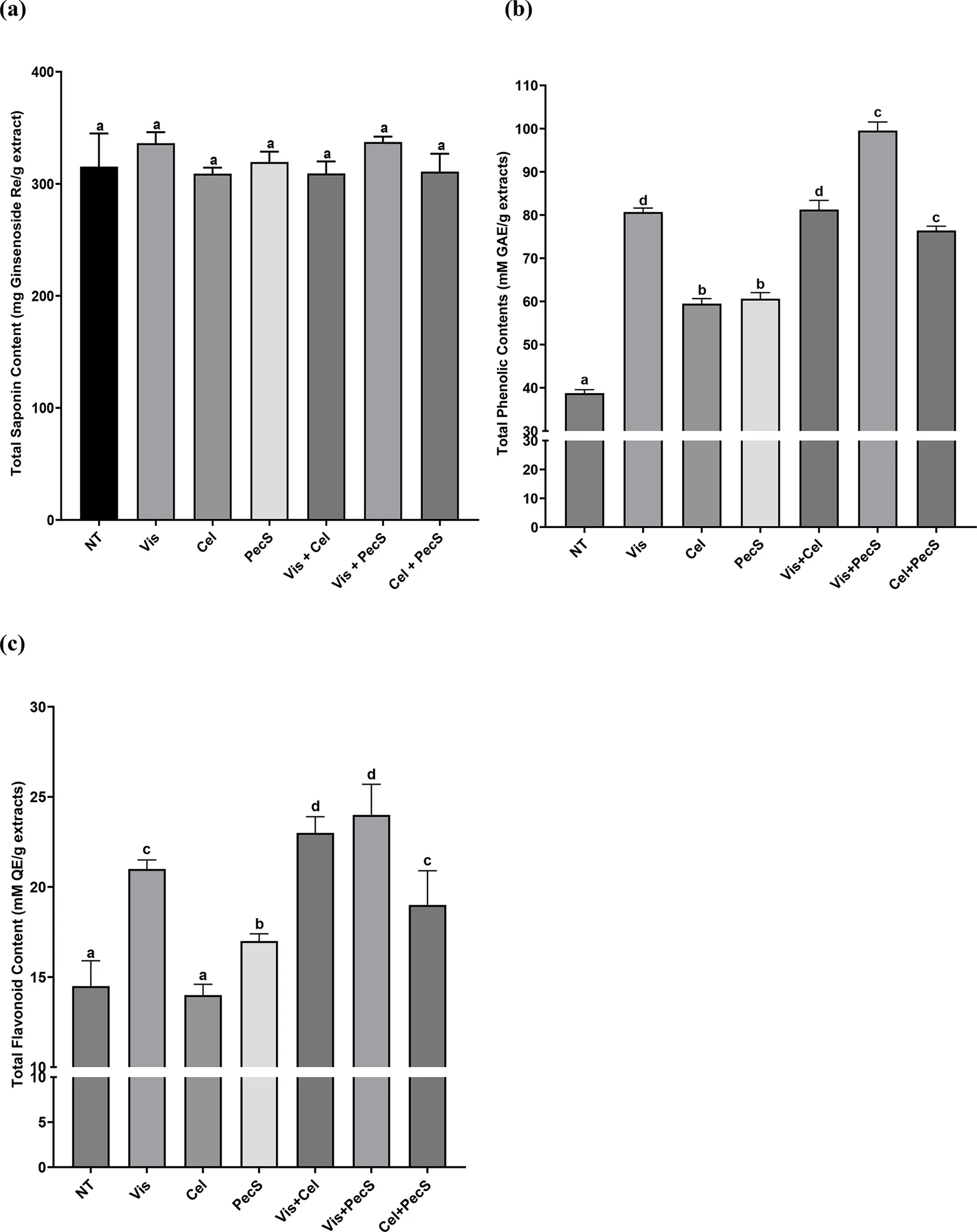
Total saponin content **a**, total phenolic content **b**, and total flavonoid content **c** of enzyme-treated WSGL. NT: non-treated; Vis, Cel, PecS: single enzyme-treated WSGL; Vis + Cel, Vis + PecS, Cel + PecS: combination of enzyme-treated WSGL. Values were expressed as means ± standard deviation (SD) of three independent experiments. Different letters indicate statistical differences by Duncan’s test

The effects of Vis, Cel, PecS, Vis + Cel, Vis + PecS, and Cel + PecS treatment on the TSC, TPC, and TFC of WSGL are illustrated in Fig. 4. As depicted in Fig. 4a, although the contents of major and minor ginsenosides in the WSGL extract varied during enzyme treatment, the TSC showed no significant differences between WSGL and enzyme-treated WSGL. The TPC of WSGL increased from 38.8 ± 0.8 to 99.5 ± 2.0 mM GAE/g extract after enzyme treatment (Fig. 4b), representing a 2.56-fold increase compared to non-treated WSGL. In comparison to the TPC of non-treated WSGL, the TPC of Vis, Cel, and PecS-treated WSGL increased by 2.08-, 1.53-, and 1.56-fold, respectively. Additionally, it was enhanced by 2.10-, 2.57-, and 1.97-fold when treated with Vis + Cel, Vis + PecS, and Cel + PecS, respectively. While the TFC showed no significant difference between non-treated WSGL and Cel-treated WSGL, the TFC of Vis- and PecS-treated WSGL increased by 1.45- and 1.18-fold, respectively (Fig. 4c). Furthermore, the TFC of Vis + Cel, Vis + PecS, and Cel + PecS-treated WSGL was 1.61-, 1.70-, and 1.36-fold higher, respectively, than that of non-treated WSGL (Fig. 4c).

### Antioxidant activity of enzyme-treated WSGL

**Fig. 5:**
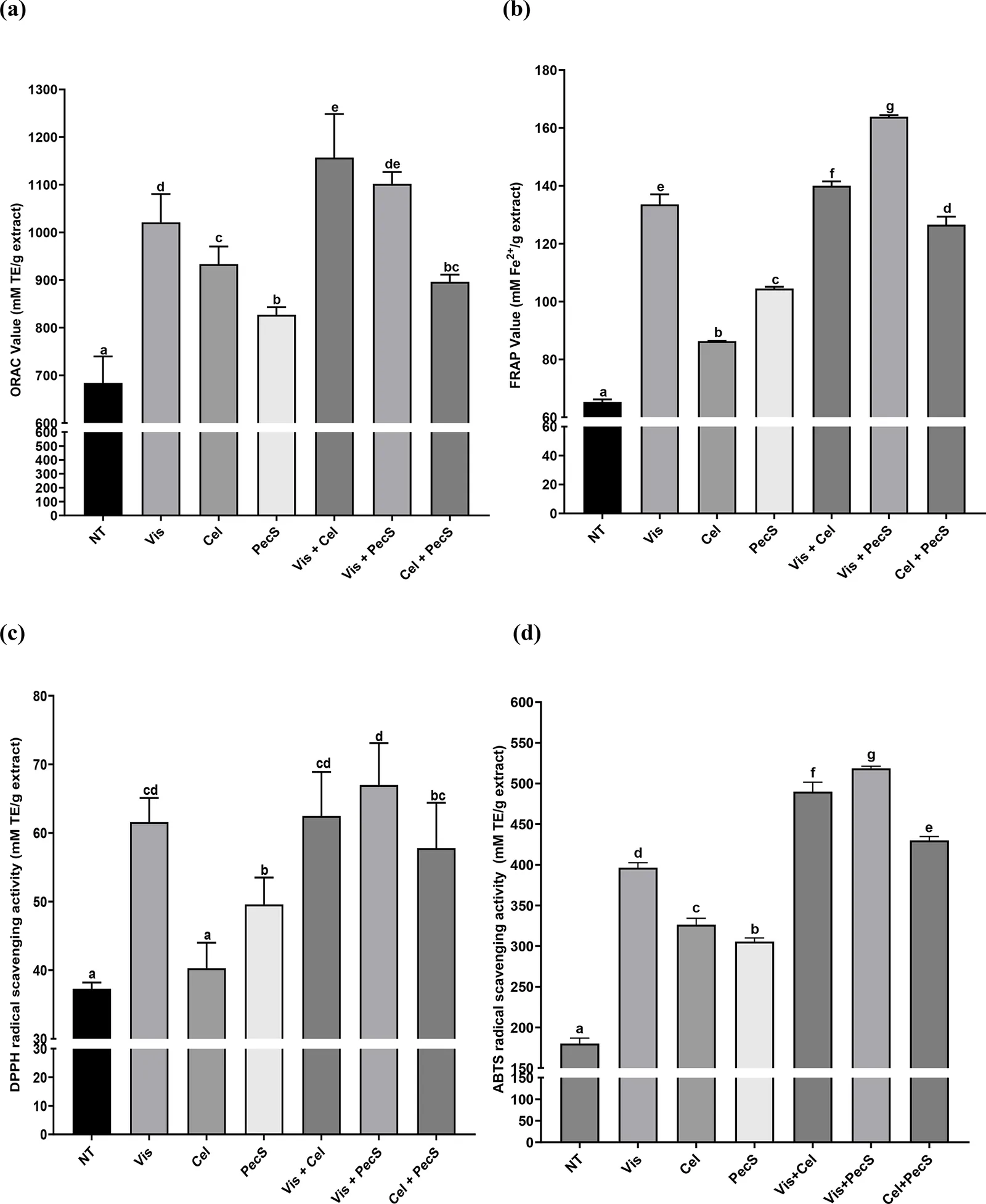
ORAC value **a**, FRAP value **b**, DPPH radical scavenging activity **c**, and ABTS radical scavenging activity **d** of enzyme-treated WSGL. Values were expressed as means ± standard deviation (SD) of three independent experiments. Different letters indicate statistical differences by Duncan’s test. NT: non-treated; Vis, Cel, PecS: single enzyme-treated WSGL; Vis + Cel, Vis + PecS, Cel + PecS: combination of enzyme-treated WSGL. Values were expressed as means ± standard deviation (SD) of three independent experiments. Different letters indicate statistical differences by Duncan’s test

The antioxidant activities of WSGL treated with enzymes, as measured by ORAC, FRAP, DPPH, and ABTS assays, are depicted in Fig. 5. The ORAC value increased from 684.1 ± 55.6 to 1157.0 ± 55.6 mM TE/g extract after enzyme treatment (Fig. 5a). Compared to non-treated WSGL, the ORAC values increased 1.49-, 1.36-, and 1.21-fold after treatment with Vis, Cel, and PecS, respectively. Furthermore, the ORAC values of WSGL treated with Vis + Cel, Vis + PecS, and Cel + PecS were 1.69-, 1.61-, and 1.31-fold higher than those of non-treated WSGL.

The FRAP value of enzyme-treated WSGL increased from 65.32 ± 0.87 to 163.85 ± 0.57 mM Fe^2+^/g extract (Fig. 5b), a 2.51-fold increase compared to non-treated WSGL. The FRAP values of WSGL treated with Vis, Cel, and PecS were increased 2.04-, 1.32-, and 1.60-fold compared to non-treated WSGL, while these enzymes in combination increased the FRAP values 2.14-, 2.51-, and 1.94-compared to non-treated WSGL. Vis + PecS-treated WSGL had the highest FRAP value. Vis treated WSGL exhibited the highest FRAP value among the three enzymes, whereas the FRAP value of WSGL treated by Vis + Cel and Vis + PecS were increased 1.11- and 1.30-fold compared to WSGL treated with Cel + PecS.

Although the DPPH radical scavenging activity did not differ significantly between Cel-treated and non-treated WSGL (Fig. 5c), those of Vis- and Cel-treated WSGL were 1.65- and 1.30-fold higher, respectively, compared to non-treated WSGL. DPPH radical scavenging activity increased from 37.3 ± 0.9 to 67.0 ± 6.1 mM TE/g extract after treatment with Vis + Cel, Vis + PecS, and Cel + PecS, a 1.80-fold increase over non-treated WSGL. Nevertheless, there was a non-significant difference in DPPH radical scavenging activity among Vis, Vis + Cel, and Cel + PecS treated WSGL. Vis + PecS-treated WSGL had the highest DPPH radial scavenging activity.

The ABTS radical scavenging activity increased from 180.2 ± 6.7 to 518.5 ± 2.9 mM TE/g extract after enzyme treatment (Fig. 5d), 2.88-fold higher than that of non-treated WSGL. The ABTS radical scavenging activity of WSGL treated with Vis, Cel, and PecS were increased 2.20-, 1.81-, and 1.70-fold, respectively, while it was enhanced 2.72-, 2.88-, and 2.39-fold by treated with combination enzymes, including Vis + Cel, Vis + PecS, and Cel + PecS-treated WSGL, respectively, compared to non-treated WSGL.

### Anti-senescence capacity of enzyme-treated WSGL

**Fig. 6:**
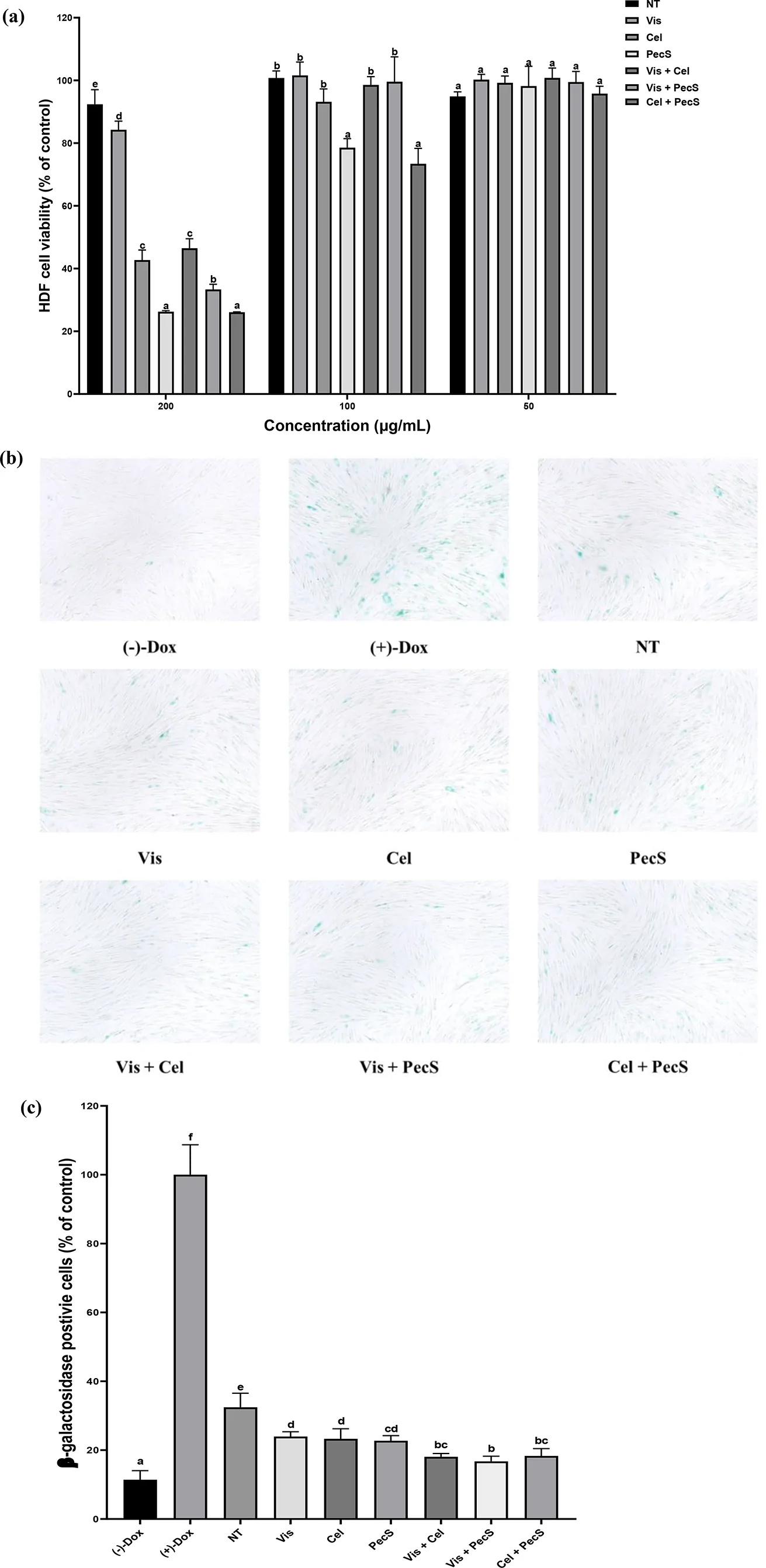
Cell viability of enzymatic biotransformation products on HDF cell **a**, senescence associated β-galactosidase positive cells stained by X-gal **b**), and suppression of senescence associated β-galactosidase positive cells by enzymatic biotransformation products **c** of enzyme-treated WSGL. NT: non-treated; Vis, Cel, PecS: single enzyme-treated WSGL; Vis + Cel, Vis + PecS, Cel + PecS: combination of enzyme-treated WSGL. Values were expressed as means ± standard deviation (SD) of three independent experiments. Different letters indicate statistical differences by Duncan’s test

The cytotoxicity of enzyme-treated WSGL at 50–200 µg/mL is shown in Fig. 6. Although non-treated and Vis-treated WSGL at 200 µg/mL maintained cell viability at over 80%, WSGL treated with Cell, PecS, Vis + Cel, Vis + PecS, and Cel + PecS at 200 µg/mL showed cell viability rates of 26.3–46.5% (Fig. 6a). Upon reducing the concentration to 100 µg/mL, the enzyme-treated WSGL maintained cell viability at > 90%. The exceptions were PecS- and Cel + PecS-treated WSGL (78.6% and 73.4% cell viability, respectively) (Fig. 6a). All samples displayed > 90% cell viability at 50 µg/mL (Fig. 6a). Thus, the effects of enzyme-treated WSGL at 50 µg/mL on the SA-β-Gal activity of HDFs were investigated and are presented in Fig. 6b–c. Micrographs of SA-β-Gal and the relative β-galactosidase activity in HDFs exposed to non-treated and enzyme-treated WSGL are shown in Fig. 6b-c. The relative β-galactosidase activity of non-treated WSGL was 32.4 ± 4.1%, compared to 16.7% for enzyme-treated WSGL.

### Inhibition of NO production by enzyme-treated WSGL

**Fig. 7:**
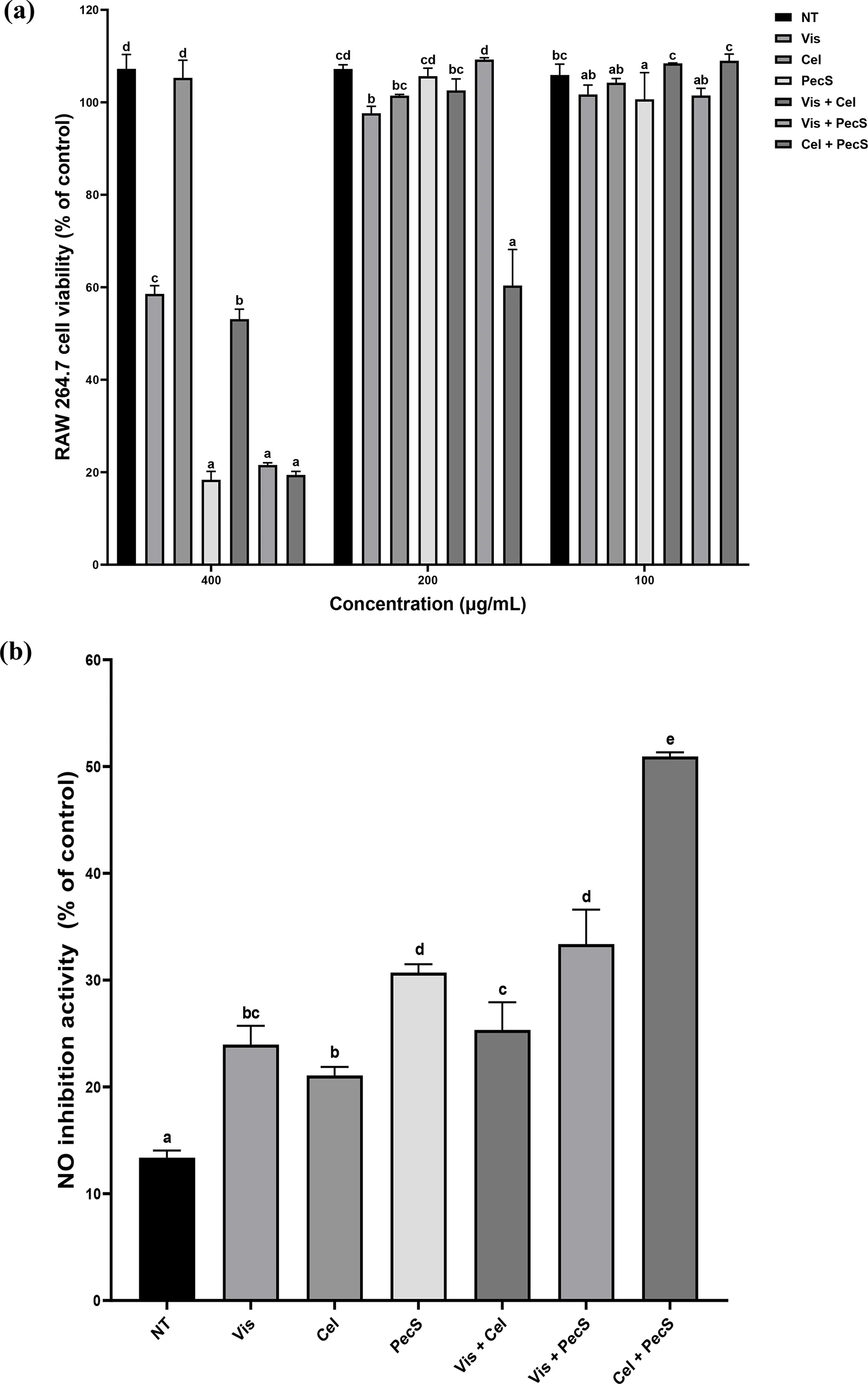
Cell viability of enzymatic biotransformation products on RAW264.7 cell **a** and measurement of nitric oxide release on RAW264.7 cell **b** of enzyme-treated WSGL. NT: non-treated; Vis, Cel, PecS: single enzyme-treated WSGL; Vis + Cel, Vis + PecS, Cel + PecS: combination of enzyme-treated WSGL. Values were expressed as means ± standard deviation (SD) of three independent experiments. Different letters indicate statistical differences by Duncan’s test

The effect of enzyme-treated WSGL on NO release by LPS-stimulated RAW264.7 cells is shown in Fig. 7. Although non-treated and Cel-treated WSGL at 400 µg/mL did not exert toxic effects on RAW 264.7 cells, Vis-, PecS, Vis + Cel, Vis + PecS, and Cel + PecS-treated WSGL at 400 µg/mL resulted in cell viability rates of 18.4‒58.6%. When the treatment concentration was reduced to 200 µg/mL, the cell viability rates of most enzyme-treated WSGL were > 95% (the exception being Cel + PecS-treated WSGL, at 60.4%). All samples showed over 90% cell viability at 100 µg/mL (Fig. 7a). Therefore, the effects of non-treated and enzyme-treated WSGL at 100 µg/mL were examined. The results are presented in Fig. 6b. The NO inhibitory activity of WSGL extract was increased from 13.4 ± 0.7% to 50.9 ± 0.4% by enzyme treatment (Fig. 7b). NO inhibitory activity did not differ significantly among Vis-, Cel-, and Vis + Cel-treated WSGL, and was 1.46-, 1.58-, and 2.42-fold higher in PecS-, Vis-PecS, and Cel + PecS-treated WSGL than Cel-treated WSGL.

## Discussion

**Fig. 8:**
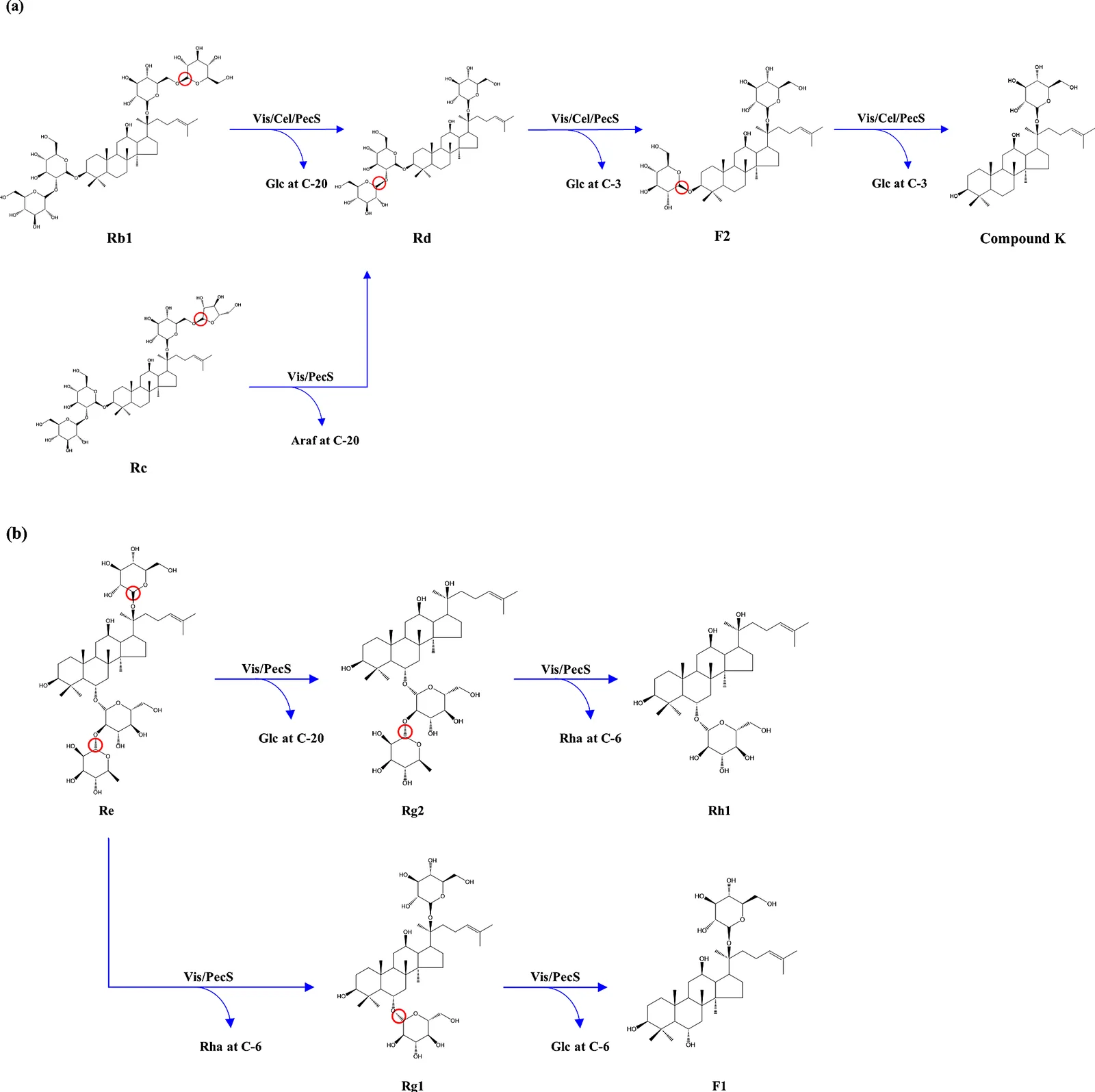
Ginsenoside bioconversion pathway: PPD-type ginsenoside **a** and PPT-type ginsenoside **b** by Vis, Cel, and PecS.

In this study, 10 ginsenosides were detected in extracted WSGL. Among them, Re accounted for 40.2% of the total extracted ginsenosides, as others reported (Lim et al. 2023; Zhang et al. 2018). Unlike ginseng extract, in which minor ginsenosides had contents of < 0.2% (Park et al. 2017), the minor ginsenosides Rg2, Rh1, F1, and F2 in WSGL and ginseng leaves account for > 10% of the total ginsenosides (Kim and Park 2019; Lim et al. 2023). Furthermore, F2 is present in raw and red ginseng at < 0.01% (Shi et al. 2013) and < 0.4%, respectively, in WSG root (Lee et al. 2020), and its content in extracted WSGL was 14.0%. However, this was lower compared to that in Hongcheon WSGL (Lim et al. 2023). The difference in F2 content between Pyeongchang WSGL and Hongcheon WSGL can be attributed to their growth in different geographical locations (Piao et al. 2020). Therefore, WSGL was selected to produce CK by enzymatic transformation in this study. Although major ginsenosides in WSGL accounted for 80.9% of the total ginsenosides, their absorption by the human intestinal tract is hampered by their low solubility, large molecular size, and poor permeability (Xu et al. 2003; Yu et al. 2012). In contrast, minor ginsenosides, especially F1, F2, and CK, are rapidly absorbed by the human intestinal tract and have enhanced cell membrane permeability and pharmaceutical activities (Hasegawa et al. 2000; Liu et al. 2015). Li et al. (2009) reported that ginsenosides with fewer glucosyl residues exerted greater anticancer effects. Moreover, the minor ginsenosides can be produced from major ginsenosides by β-glucosidase (Song et al. 2017; Choi et al. 2014). The food-grade commercial enzymes, including Vis, Cel, and PecS, have β-glucosidase activity (Andrades et al. 2019; Kim et al. 2019) and hydrolyze the β-glycosidic linkage in major ginsenosides to produce minor ginsenosides (Upadhyaya et al. 2016; Wang et al. 2021). Therefore, this study aims to enrich CK in WSGL by using commercial enzymes and their combination and the effects of WSGL-enriched CK on the biochemical properties investigated. The extracted WSGL contained ginsenosides, glucose, fructose, and sucrose. Xiao et al. (2004) reported that an increase in glucose content significantly inhibits β-glucosidase and cellulase activities (Xiao et al. 2004). Andrades et al. (2019) showed that PecS and Cel were inhibited by 90.21 and 107.29 mM glucose, respectively. Therefore, glucose, fructose, and sucrose in extracted WSGL were removed by yeast bead immobilization. In this study, we found that Vis-treated WSGL showed complete hydrolysis or decreased contents of ginsenoside Rb1, Rc, Rd, Re, Rg2, and Rg3, while the contents of Rh1, F1, and F2 were increased compared to non-treated WSGL. Similarly, in Vis-treated red ginseng extract, the Rb1 content was decreased 10.36-fold, and the Rd and F2 contents were increased 2.41- and 5.02-fold after treatment with Vis L, whereas only a small amount of CK was produced (Park et al. 2021). However, these results differed in Vis-treated red ginseng powder (Kim et al. 2019). Kim et al. (2019) reported that Vis-treated red ginseng powder showed increased Rg3, Re, and Rb1 contents (Kim et al. 2019). Cel-treated WSGL exhibited no significant changes in Rc, Rd, Re, Rg1, Rg2, Rg3, Rh1, and F1 contents, while Rb1 was completely removed. The content of F2 was decreased to 2.4 ± 0.3 mg/g extract, but CK was increased to 15.1 ± 0.2 mg/g extract. Lee et al. (2012) reported that enzyme treatment of Cel-treated ginseng leaves reduced the ginsenoside contents. The extracted red ginseng had a 32.49-fold higher Rb1 content than extracted-WSGL but lower Rd and F2 contents. Park et al. (2021) reported that the treatment of red ginseng extract using Cel resulted in the hydrolysis of most of the Rb1 to Rd, and a small amount of CK was formed (0.19 mg/g). The Ck content in Cel-treated WSGL was 61.05 and 79.47-fold higher than in Cel-treated red ginseng extract (Park et al. 2021). WSGL treated with PecS showed complete hydrolysis of Rb1 and decreased content in Rc, Rd, Rg1, and F2 compared to non-treated WSGL (Fig. 2a-c, e, j). On the other hand, the content of Rh1 and F1 increased 1.32‒1.97-fold after 48 h compared to non-treated WSGL (Fig. 2h, i). Furthermore, the highest content of CK was observed in PecS-treated WSGL. Mok et al. (2023) reported a non-significant difference in total ginsenoside content between control and PecS-treated WSG. However, the contents of minor ginsenosides (Ro, Rh1, F2, CO, Mc1, and Rg3) in PecS-treated WSG were increased compared to the control, and CK was not detected (Mok et al. 2023). The higher contents of minor ginsenosides, including Rh1, F1, F2, and CK, in enzyme-treated WSGL than enzyme-treated red ginseng, ginseng leaves, and WSG (Kim et al. 2019; Lee et al. 2012; Mok et al. 2023) may be a result of inhibition of the enzymes by fructose, glucose, and sucrose in red ginseng, ginseng leaves, and WSG (Andrades et al. 2019). Furthermore, Vis hydrolyzed Rb1, Rc, Rd, and Re in WSGL extract to produce F1, F2, and a small amount of CK, whereas PecS hydrolyzed Rb1, Rc, Rd, Re, and Rg1 in WSGL extract to produce F1, F2, and CK. In addition, Cel hydrolyzed Rb1 and F2 in WSGL extract to produce CK. We propose biotransformation pathways of minor from major ginsenosides in Fig. 8. For PPD-type ginsenoside, the hydrolytic pathways are Rb1 and Rc → Rd → F2 → CK for Vis- and PecS-treated WSGL and Rb1 → Rd → F2 → CK for Cel-treated WSGL (Fig. 8a). For PPT-type ginsenosides, the hydrolytic pathways are Re → Rg2 → Rh1 and Re → Rg1 → F1 by Vis- and PecS-treated WSGL (Fig. 8b). Additionally, the combination of enzymes on CK production in WSGL was investigated. Vis + Cel, Vis + PecS, and Cel + PecS combination increased the F1 and CK content in WSGL. These results are consistent with the report by Park et al. (2021) that the production of F2 and CK in red ginseng extract was increased by treatment with a combination of enzymes.

In this study, we observed that major ginsenosides were converted into minor ginsenosides during enzyme treatment. However, the TSC showed no significant difference between non-treated WSGL and enzyme-treated WSGL. In contrast, the TPC and TFC of WSGL increased following enzyme treatment (Fig. 4b, c). Ma and Cheung (2007) reported that an increased number of hydroxyl groups could contribute to the elevated polyphenol content measured by the Folin-Ciocalteu method. Additionally, Shraim et al. (2021) noted that flavonoids with more hydroxyl groups have greater binding affinity to metal ions like Al^3+^. Therefore, the increase in TPC and TFC of WSGL after enzyme treatment might be attributed to the hydrolysis of glycosidic linkages by enzymes, resulting in the exposure of hydroxyl groups in the phenolic and flavonoid compounds.

**Fig. 9:**
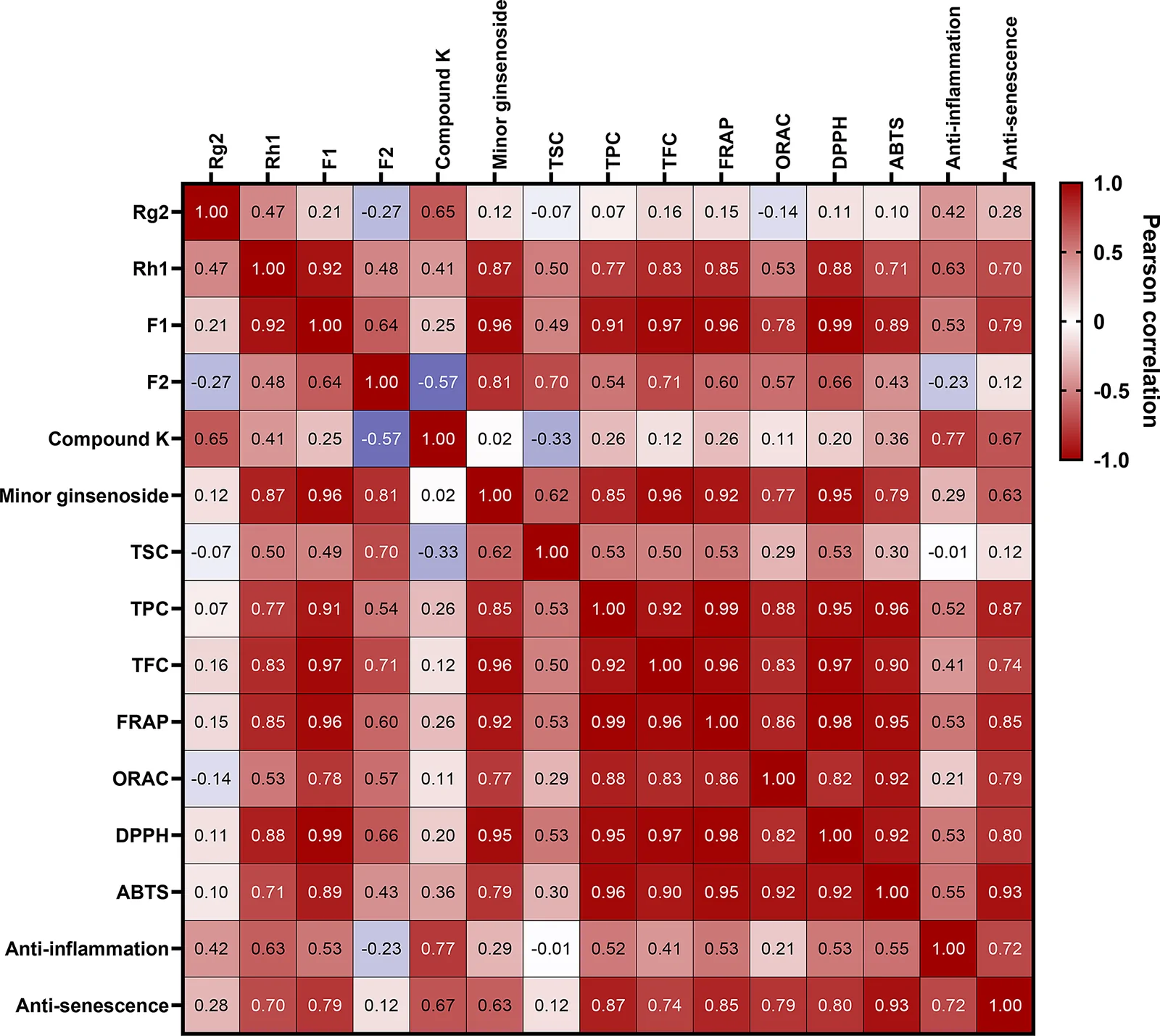
Heatmap of pearson correlation coefficient

Measurement of antioxidant capacity by ORAC, FRAP, DPPH, and ABTS methods exhibited enzyme treatment of WSGL increased antioxidant capacity compared to non-treated WSGL. Furthermore, treatment of WSGL with the combination of enzymes has higher antioxidant capacity than single enzyme treatment of WSGL. Chen et al. (2020) reported that aglycones have stronger antioxidant activities than glycosides because the free hydroxyl group of phenolics acts as a radical scavenger. Thus, the elevated antioxidant activity in enzyme-treated WSGL can be attributed to the transformation of major into minor ginsenosides by carbohydrate-cleaving enzymes. These results are consistent with the report by Lee et al. (2012) that the antioxidant activity of ginseng leaves was enhanced by treatment with carbohydrate-cleaving enzymes, such as pectinase, polygalacturonase, and β-glucanase, as a result of increased levels of polyphenols and flavonoids (Lee et al. 2012). Similarly, Park et al. (2021) showed that the enhanced DPPH and ABTS radical scavenging activity of enzyme-treated red ginseng resulted from the elevated CK and polyphenol contents. A Pearson correlation test to verify the relationship indicated TPC is very highly correlated with ORAC (*r* = 0.88), FRAP (*r* = 0.99), DPPH (*r* = 0.95), and ABTS (*r* = 0.96), while TFC was very highly correlated with ORAC (*r* = 0.83), FRAP (*r* = 0.96), DPPH (*r* = 0.97), and ABTS (*r* = 0.90). Although both CK and F1 positively correlated with antioxidant activity, F1 displayed a stronger correlation than CK (Fig. 9).

Senescence refers to the gradual deterioration of cellular functions in response to stress or damage (McHugh and Gil 2018). Oxidative stress, inflammation, DNA damage, and mitochondrial dysfunction can induce senescence (Dodig et al. 2019). Senescence-associated β-galactosidase (SA-β-Gal) stains senescent cells (Lee et al. 2006). There was a non-significant difference in β-galactosidase activity among Vis-, Cel-, and PecS-WSGL and combination enzyme-treated WSGL. However, HDFs exposed to WSGL treated with enzyme combinations showed lower levels of β-galactosidase. This may be attributed to the increased levels of minor ginsenosides and antioxidant activities in the enzymatic biotransformation products (Kirsch et al. 2022; Piechota et al. 2016). Oxidative stress and ROS are linked to senescence, and SA-β-Gal expression responds to oxidative stress. The minor ginsenoside F1, the content of which was significantly enhanced by enzymatic biotransformation, has been reported to suppress senescence by downregulating the secretion of senescence-associated secretory phenotype markers, including IL-6 and IL-8 (Hou et al. 2018). By contrast, CK suppresses senescence-associated phenotypes, such as iNOS expression (Sharma and Lee 2020). Park et al. (2005) reported that CK significantly inhibits the expression of cyclooxygenase-2 (COX-2), the product of which modulates the senescence-associated secretory phenotype. A Pearson correlation analysis showed F1 (*r* = 0.79) and CK (*r* = 0.67) are highly correlated with anti-senescence capacity (Fig. 9).

Inflammation bolsters the immune response to harmful stimuli, such as pathogens, damaged cells, and toxic compounds. However, chronic or uncontrolled inflammation can lead to complications, including autoimmune diseases. NO, a compound central to the onset of inflammatory diseases, is a marker of anti-inflammatory activity (Chen et al. 2017). PecS-, Vis + PecS, and Cel + PecS-treated WSGL had higher NO inhibitory activities than Vis-, Cel-, and Vis + Cel-treated WSGL (Fig. 8b). The findings suggest that CK content influences NO inhibitory activity. Indeed, Ryu et al. (2018) reported that CK inhibits NO production by repressing iNOS in a concentration-dependent manner. Furthermore, Huynh et al. (2020) showed that Rg2 and Rh1 inhibit LPS-induced NO production. A Pearson correlation test to verify the relationship exhibited CK is strongly correlated with anti-inflammatory capacity (*r* = 0.77) (Fig. 9).

In conclusion, we produced the minor ginsenosides Rh1, F1, F2, and CK from major ginsenosides in WSGL extract using commercial enzymes, individually and in combination. The CK, F1, and F2 contents in WSGL were 25.9 ± 1.0, 55.1 ± 1.7, and 19.9 ± 0.8 mg/g after treatment with Cel + PecS, Vis, and Vis + Pec, respectively. Enzyme-treated WSGL had high antioxidant and anti-inflammatory activities, and inhibited senescence, suggesting its potential for use as a raw material in the food and pharmaceutical industries.

## Electronic supplementary material

Below is the link to the electronic supplementary material.

Supplementary Material 1

## Data Availability

All data generated or analyzed during the current study are available from the corresponding author upon reasonable request.
