# Supplementary material for: Enzymatic upcycling of wild-simulated ginseng leaves for enhancing biological activities and compound K
Source: Appl Microbiol Biotechnol. 2024 Feb 14;108:207. doi: 10.1007/s00253-024-13028-2 (PMC10866779; doi:10.1007/s00253-024-13028-2)
Supplement: Supplementary file 1 [file 253_2024_13028_MOESM1_ESM.pdf]

Applied Microbiology and Biotechnology

Supplementary materials

Enzymatic Upcycling of Wild-Simulated Ginseng Leaves for Enhancing Biological Activities and Compound K

Juho Lim[1], Hayeong Kim[2*], Gha-hyun J. Kim[3], Taeyoon Kim[1], Choon Gil Kang[4], Seung Wook Kim[4], Doman Kim[1, 2, 5*]

[1]Graduate School of International Agricultural Technology, Seoul National University, Pyeongchang-gun, Gangwon-do, 25354, Republic of Korea.

[2]Institute of Food Industrialization, Institutes of Green Bioscience & Technology, Center for Food and Bioconvergece, Seoul National University, Pyeongchang-gun, Gangwon-do, 25354, Republic of Korea.

[3]Department of Bioengineering and Therapeutic Sciences and Programs in Biological Sciences and Human Genetics, University of California San Francisco, San Francisco, CA, 94158, USA

[4]Ottogi Corporation, Anyang-si, Gyeonggi-do, 14060, Republic of Korea.

[5]Fervere Campus Corporation, Pyeongchang-gun, Gangwon-do, 25354, Republic of Korea

*** Correspondence to: Doman Kim**

Email: kimdm@snu.ac.kr; Tel: +82-33-339-5720; Fax: +82-33-339-5716

*Co-corresponding Author: Hayeong Kim

E-mail: hara2910@snu.ac.kr. Tel:+82-33-339-5736

Table s1. UPLC-QDa mass condition of standard sample

Compound	Concentration range (μg/mL)	M/Z	Polarity	Cone voltage (V)	Capillary voltage (kV)	Linearity (R^2)	Regression equation
**Rb1**	**0.1 – 20.0**	**1132.0**	**Positive**	**10**	**1.5**	**0.999**	**Y=58400X-32100**
**Rc**	**0.2 – 20.0**	**1101.6**	**Positive**	**35**	**1.5**	**0.999**	**Y=150000X+8180**
**Rd**	**0.1 – 20.0**	**969.7**	**Positive**	**20**	**1.5**	**0.999**	**Y=114000X-69100**
**Re**	**0.1 – 20.0**	**945.6**	**Negative**	**45**	**0.8**	**0.998**	**Y=46200X-8860**
**Rg1**	**0.1 – 10.0**	**823.6**	**Positive**	**25**	**1.5**	**0.995**	**Y=108000X+85400**
**Rg2**	**0.1 – 20.0**	**783.6**	**Negative**	**40**	**0.8**	**0.999**	**Y=95100X+1770**
**Rg3**	**0.8 – 20.0**	**807.6**	**Positive**	**50**	**1.5**	**0.997**	**Y=92500X-12700**
**Rh1**	**0.1 – 20.0**	**683.5**	**Negative**	**20**	**0.8**	**0.999**	**Y=113000X-22100**
**F1**	**0.1 – 20.0**	**661.6**	**Positive**	**25**	**1.5**	**0.999**	**Y=229000X+14800**
**F2**	**0.1 – 10.0**	**829.7**	**Negative**	**10**	**0.8**	**0.994**	**Y=69400X+39000**
**Compound K**	**0.2 – 10.0**	**645.5**	**Positive**	**45**	**1.5**	**0.997**	**Y=341000X+212000**

Table s2. List and characteristic of commercial enzymes

No.	Commercial name	Commercial sources	Source	Enzyme activity	Enzyme unit	pH	Temperature (°C)
**1**	Viscozyme L	Novozymes, Bagsvaerd, Denmark	*Aspergillus aculeatus*	β-glucanase, cellulase, xylanase, hemicellulase	100 FBG/mL	3.5-5.5	25-55
**2**	Celluclast 1.5L		*Trichoderma reesei*	Cellulase	700 EGU/mL	4.0 - 6.0	45-65
**3**	Pectinex Ultra SP-L		*Aspergillus aculeatus*	Polygalacturonase	3,800 PGNU/mL	4.0 - 5.0	40-60

FBG fungal beta-glucanase unit; *EGU* endo glucanase unit; *PGNU* polygalacturonase unit
